# Effectiveness of Sedoanalgesia in Percutaneous Liver Biopsy Premedication

**DOI:** 10.5005/jp-journals-10018-1236

**Published:** 2017-09-29

**Authors:** Orhan Sezgin, Serkan Yaras, Fehmi Ates, Engin Altintas, Bunyamin Saritas

**Affiliations:** 1 Department of Gastroenterology, Mersin University, Mersin, Turkey

**Keywords:** Gastroenterology, Hepatitis B, Hepatology, Viral hepatitis.

## Abstract

**Aim:**

Percutaneous needle liver biopsy (PLB) is frequently associated with pain and anxiety. This may discourage the patients for biopsy, and rebiopsies, if needed. We planned a study to investigate the efficacy of additional analgesia or sedation for PLB.

**Materials and methods:**

The study has been designed as a single-center, prospective study. The PLB was planned for 18- to 65-year-old consecutive patients who were included in the study. The patients were divided into three premedication groups as control, Meperidine, and Midazolam. Hospital Anxiety and Depression Scale (HADS) was used to measure each subject’s anxiety level. Fifteen minutes before the biopsy, 1 mL 0.9% NaCl subcutaneously (sc), 1 mg/kg (max 100 mg) Meperidine sc, or 0.1 mg/kg (max 5 mg) Midazolam intravenously was administered to patients respectively. Then PLB was done with 16 G Menghini needle. The day after, the patients were asked about feelings regarding biopsy.

**Results:**

Groups were similar by gender and age. The HADS scores prior to PLB and on visual analog scale (VAS, 1-10 points) score during PLB were similar. In the three groups, 7, 12, and 7 patients, respectively, experienced no pain. Other patients explained pain as mild or moderate or severe. The number of patients who agreed for possible rebiopsy was higher in Meperidine and Midazolam groups than in the control group.

**Conclusion:**

Premedication with Meperidine or Midazolam in PLB would improve patients’ tolerance, comfort, and attitude against a possible repeat PLB.

**How to cite this article:** Sezgin O, Yaras S, Ates F, Altintas E, Saritas B. Effectiveness of Sedoanalgesia in Percutaneous Liver Biopsy Premedication. Euroasian J Hepato-Gastroenterol 2017;7(2):146-149.

## INTRODUCTION

Although noninvasive alternatives such as transient elas-tography and the fibro test have partly been superseded, PLB is still a gold standard method for the diagnosis of liver disease and the response to treatment. There is no consensus on analgesic and/or sedative premedication for PLB, in terms of the agents to be used and cost-effectiveness. In our daily practice, we use either local anesthesia alone or Meperidine and Midazolam additionally, for PLB premedication. The biopsy procedure is often frightening and uncomfortable for the patient. Such experiences can discourage patients for the follow-up biopsies in the future. It sounds logical that if the biopsy procedure is comfortable, the biopsy and repeat biopsy if needed would be more acceptable by the patients. We designed a study to evaluate the effectiveness of analgesia and sedation in addition to local anesthesia.

## MATERIALS AND METHODS

The study was designed as a single-center study. Neither practitioner nor the patient who underwent the procedure knew the premedication. In our gastroenterology clinic, all patients who accepted and met the inclusion and criteria were consecutively invited and included in the study. All patients were informed about the need for PLB, the technical process, and possible complications. The patients were consecutively divided into three groups: Control (saline), Meperidine, and Mid-azolam, without knowledge of biopsy practitioner and the patients. Participants were requested to complete a demographic and general health questionnaire, as well as the Spielberger Anxiety Questionnaire, which was used to measure each subject’s state anxiety level approximately 2 hours before the biopsy.^1^ A detailed explanation on how to use the 10-cm blank VAS was given to all subjects by one investigator (SY). Premedication was applied in a double-dummy pattern, resembling each other in all groups. The study was approved by the Mersin University Pharmaceutical Research Commission, decree B.30.2.MEU.0.20.05.04./70, dated Mar 16, 2011. Liver biopsy procedures were managed by the same practitioner, experienced for PLB (SY), during the study period. Written informed consent was obtained from patients. Standard percutaneous liver biopsy is performed on patients fasted overnight, in the same position: Lying on the back, right arm under the head, and then by transabdominal ultrasonography, the most appropriate entry location is detected in the skin and marked.^2^ The biopsy site was located with bedside ultrasonography usually in the seventh or eighth intercostal space in the midaxillary line. Before the procedure, pretreatment agents were given according to the patient group, without the knowledge of the practitioner. Then place of biopsy was cleaned with povidone-iodine followed by skin and sc anesthesia with 10 mL prilocaine HCl (Citanest, Astra Zeneca). Then, PLB was performed with the standard 16 gauge disposable Menghini needle aspiration. After the biopsy procedure, the patient then was made to lie on his/her right side for 1 hour and then supine for 4 hours and pulse and blood pressure were monitored regularly in order to detect complications early. Then, patients without any problem were discharged. The next day the patients came to the clinic, and were evaluated with ultrasound for any subcapsular, intrahepatic, or peritoneal bleeding. All patients were questioned with a questionnaire if there was a state of anxiety prior, during, and after the procedure and thoughts about their pain and the biopsy procedure. A 10-cm blank VAS was used to grade the intensity of the subjects’ pain. Subjects were asked to mark their pain intensity on this scale, with 0 indicating no pain and 10 indicating the worst imaginable pain. Spielberger Anxiety Questionnaire was used to measure each subject’s anxiety level before the biopsy. Participants were requested to complete the HADS^3^ to state the level of anxiety before biopsy. The results obtained were used for statistical analysis. In addition, patients were asked whether they remembered the PLB process, to evaluate retrograde amnesia related to Midazolam.

## STATISTICAL ANALYSES

The data from Meperidine, Midazolam, and the control groups were analyzed with Shapiro-Wilk test to check whether they were in normal distribution. For parameters with normal distribution, descriptive statistics were given such as mean and standard deviation. For parameters which were inconsistent with normal distribution, median and percentile values were given. Number and percentage values were given as descriptive statistics for categorical parameters. Mann-Whitney U test was used to compare all three groups, about mean values of pain which the patient perceived during the biopsy and the day after. Kruskal-Wallis test was used to compare the groups with the mean values of pain the patient perceived during the biopsy and the day after. Student’s t-test was used to compare the average length of biopsy material between the patients. The average age differences between groups were analyzed with variance analysis. The proportional differences between the two groups, if any, were analyzed with the Z test. Chi-square analysis was used for determining the relationship between categorical variables.

## RESULTS

A total of 90 patients were enrolled in the study, with 30 patients in each group. There were 62 males, 28 females with mean age of 41.63 years. The average age, sex distribution, education, and PLB indications were not different between the groups. There was no difference between the groups on anxiety level (HADS score) before the PLB. Totally 11/90 patients (12.2%) were very anxious and even thought of giving up the process a few times. Remaining 28/90 patients (31.1 %) were anxious and 51/90 patients (56.7%) did not feel worried. Three groups were similar in answer to the question: "How did you feel during the PLB?" Most of the patients were observed to be relaxed during the process. There were no differences between the three groups on the pain (VAS score) during the PLB. In our study, there were pain due to biopsy in 71.1% of patients (61.0% mild, 39.0% medium or heavy). The pain was located on the abdomen (4, 1, and 4 patients in control, Meperidine, and Midazolam groups, respectively) or radiating to the right shoulder (2, 5, and 6 patients in control, Meperidine and Midazolam groups respectively). No patient stated severe pain. There was no pain requiring hospitalization. The average length of tissues achieved during PLB was 23 ± 9 mm and the length of the liver tissue was not associated with pain level. The conceptions of patients about the process, after the process, patients stating the process difficult accounted for 20% of the control group, 6.7% of the Meperidine group and 6.6% of Midazolam group respectively. Although more patients in the control group had stated the process as difficult than the patients in the other groups, there was no statistical difference between the groups. There were differences between the control group and both the Meperidine and Midazolam groups on the answer to the question for their attitude about the repeat biopsy. The rates of the patients who said he/she would accept the repeat biopsy without thinking were 33% in control group, 53% in Meperidine group, and 50% in Midazolam group. The difference was statistically significant (p < 0.035). The rates of those a bit worried about the process were 50, 30, and 33%, respectively (p < 0.035). The rates of the patients who said he/ she would reject the rebiopsy were 13.3% in control group, 6.7% in Meperidine group, and none in the Midazolam group. There were no major complications or side effects observed during PLB procedures.

## DISCUSSION

Percutaneous needle liver biopsy is a fundamentally important procedure in the evaluation of chronic and acute liver diseases.^3^ Liver biopsy is frequently associated with pain and anxiety. Moderate to severe pain, often requiring hospitalization, is seen in 1 to 5% of patients in the past series.^4,5^ Despite its prevalence and intensity, little has been done to evaluate the pain associated with liver biopsy or its treatment, so far. The procedure is usually performed under local anesthesia only. Mild anxiolytic treatment plus local anesthetic infiltration seem to produce insufficient analgesia, thus indicating that a more profound analgesic treatment is required for better control of this pain.^6^

Meperidine is a phenylpiperidine derivative opiate analgesic with its analgesic effects found by chance in 1939. Meperidine 75 to 100 mg given sc causes analgesia equal with 10 mg morphine given in the same way. There is also a sedating effect. Duration of action is shorter than that of morphine; elimination half-life is 3 to 4 hours. It is widely used in particular in musculoskeletal pain, for headache relief, cancer pain, lumbar disc herniations, all kinds of postoperative pain, applications of sedation, and analgesia.^7^

Midozolam is a short-acting, water-soluble benzodi-azepine drug that acts similarly to diazepam on y-amino butyric acid-associated benzodiazepine receptors.^8^ It has anxiolytic, sedative, hypnotic, anticonvulsant, muscle-relaxant, and anterograde amnesic effects. Its chemical structure is different from classic benzodiazepines such as diazepam, and this is responsible for its unique characteristics of rapid absorption and rapid metabolism.^9^

Efficacy and safety of diazepam and Meperidine combination were investigated in pediatric patients for gastrointestinal procedures such as colonoscopy, endos-copy, and liver biopsy and concluded that it is an effective and reliable method in patients older than 6 months.^10^

In the literature, very few studies were aimed at the prevention of pain and anxiety due to liver biopsy. In those studies, the goals and results were different. The use of Midazolam in PLB was studied by Brouillette et al^11^ in 41 patients totally (21 with 2 mg Midazolam before and after biopsy, 20 patients with placebo). They concluded that Midazolam administration before and after biopsy procedure reduces the discomfort of the patients during the biopsy procedure and improves compliance of the patients in the necessity of repeat biopsy. Our study reveals this conclusion. The rates of the patients who said they would reject the rebiopsy were 13.3% in control group, 6.7% in Meperidine group, and none in the Midazolam group. Midazolam could be more convenient than Meperidine because of its anterograde amnestic effect. Interestingly, our patients mostly remembered the process, even in the Midazolam group.

In another study, efficacy and safety of the premedication for liver biopsy were investigated in patients with the lysosomal storage disease under 16 years old. A total of 30 patients were included in the study and oral chlorpromazine, Meperidine, and pentobarbital premedication were administered to the patients. Authors concluded that liver biopsy can be performed safely with appropriate patient selection and adequate sedation.^12^ We hypothesized which drug (Midazolam or Meperidine) is more effective for premedication. In this context, there were no differences between both the drug groups about the pain level, and the attitude for rebiopsy. There was no patient who regretted rebiopsy in the Midazolam group. This result may be in favor of premedication with Midazolam.

Furthermore, according to Eisenberg^13^ and Sherlock and Dooley,^14^ "sedation is not given routinely before biopsy as it may interfere with the patient’s cooperation." Schiff and Schiff^15^ stated that "it is not necessary to premedicate the patient before the biopsy." Thus, not unexpectedly, a nationwide survey in France showed that sedation or premedication was given in only 46% of 2,084 biopsies.^16^

Moreover, there are no precise guidelines from international liver societies and the recommendations are different. By American Association for the Study of Liver Diseases guidelines, use of oral or intravenous anxiolytic therapy or conscious sedation is variable; available data indicate that it is safe when used.^17^ Midazolam sedation for the biopsy procedure should be given to anxious patients in accordance with the British Society of Gas-troenterology guidelines, if there is no contraindication. Midazolam should be given with caution in the context of liver disease.^18^ There is no comment about premedication in Canadian guidelines.

In our clinic, percutaneous liver biopsy frequently was applied for patients with viral hepatitis. In an earlier study, we had evaluated retrospectively the ultrasound-assisted liver biopsy procedures of 784 patients in our clinic. In that study, we had found that totally 21.8% of patients stated pain during the process.^19^

Accordingly, we estimated that it was interesting to perform a trial comparing the action of Meperidine, Midazolam, and placebo on anxiety, pain, and acceptability of percutaneous liver biopsy. Based on the earlier studies and our own experience, this study was designed to investigate the effects of premedication with Meperi-dine or Midazolam on the pain and anxiety level of the patients who underwent biopsy and the overall acceptability of biopsy by them. In our study, there were pain due to biopsy in 71.1% of patients (61.0% mild, 39.0% medium or heavy). The pain was located on the puncture site, and abdomen, or radiated to the right shoulder. No patient stated severe pain. There was no pain requiring hospitalization. There was anxiety in 73.3% of patients before the biopsy process, in our study. Anxiety can interfere with patient compliance, hence the success of process.

In this study, we used HADS scores of patients for anxiety assessment. The HADS score system was originally written in English language, and has not been validated in Turkish language so far. There are many studies comparing the HADS score system in other languages apart from English, which concluded some difficulties.^20^ We realized no major difficulty about patients’ understanding of the test during the study.

Our observations during PLB procedures showed that patients’ moods during the biopsy may be quite variable. Some patients can easily tolerate the biopsy procedure only with local anesthesia, some can experience the procedure very irritated and painfully. Though these findings existed at the time, we realized that attitudes may change over time and the patient could change their behavior. Therefore, it sounds like, more the patient comfort, more courageous he/she will be in the next biopsy. Here an important restrictive point may be the addictive properties of both drugs.

Another interesting finding of our study was that, although there was no difference about the level of the pain in three groups, the attitude for possible rebiopsy was different in groups favoring the use of premedication.
